# Diagnostic performance of allele-specific RT-qPCR and genomic sequencing in wastewater-based surveillance of SARS-CoV-2

**DOI:** 10.1016/j.eehl.2025.100135

**Published:** 2025-01-21

**Authors:** Md Pervez Kabir, Élisabeth Mercier, Walaa Eid, Julio Plaza-Diaz, Patrick M. D'Aoust, Chrystal Landgraff, Lawrence Goodridge, Opeyemi U. Lawal, Shen Wan, Nada Hegazy, Tram Nguyen, Chandler Wong, Ocean Thakali, Lakshmi Pisharody, Sean Stephenson, Tyson E. Graber, Robert Delatolla

**Affiliations:** a Department of Civil Engineering, University of Ottawa, Ottawa, Ontario, Canada; b Children's Hospital of Eastern Ontario Research Institute, Ottawa, Ontario, Canada; c Division of Enteric Diseases, National Microbiology Laboratory, Public Health Agency of Canada, Winnipeg, Manitoba, Canada; d Canadian Research Institute for Food Safety, Department of Food Science, University of Guelph, Guelph, Ontario, Canada

**Keywords:** SARS-CoV-2 variants, Haplotype, Diagnostic sensitivity, Diagnostic specificity, Youden's index

## Abstract

Clinical genomic surveillance is regarded as the gold standard for monitoring SARS-CoV-2 variants globally. However, as the pandemic wanes, reduced testing poses a risk to effectively tracking the trajectory of these variants within populations. Wastewater-based genomic surveillance that estimates variant frequency based on its defining set of alleles derived from clinical genomic surveillance has been successfully implemented. This method has its challenges, and allele-specific (AS) RT-qPCR or RT-dPCR may instead be used as a complementary method for estimating variant prevalence. Demonstrating equivalent performance of these methods is a prerequisite for their continued application in current and future pandemics. Here, we compared single-allele frequency using AS-RT-qPCR, to single-allele or haplotype frequency estimations derived from amplicon-based sequencing to estimate variant prevalence in wastewater during emergent and prevalent periods of Delta, Omicron, and two sub-lineages of Omicron. We found that all three methods of frequency estimation were concordant and contained sufficient information to describe the trajectory of variant prevalence. We further confirmed the accuracy of these methods by quantifying the diagnostic performance through Youden's index. The Youden's index of AS-RT-qPCR was reduced during the low prevalence period of a particular variant while the same allele in sequencing was negatively influenced due to insufficient read depth. Youden's index of haplotype-based calls was negatively influenced when alleles were common between variants. Coupling AS-RT-qPCR with sequencing can overcome the shortcomings of either platform and provide a comprehensive picture to the stakeholders for public health responses.

## Highlights

• SARS-CoV-2 variant frequency estimation in wastewater is comparable between AS-RT-qPCR and genomic sequencing.
• Diagnostic performance confirms the accuracy of AS-RT-qPCR and genomic sequencing for variant estimation in wastewater.
• Diagnostic performance of AS-RT-qPCR is reduced during periods of lower SARS-CoV-2 variant concentration in wastewater.
• Diagnostic performance of wastewater sequencing is reduced due to insufficient sequencing depth of the targeted allele.
• Monitoring of more than one allele is suggested to overcome limitations of both AS-RT-qPCR and sequencing.
• Accuracy of variant frequency estimation by sequenced haplotype requires careful curation.

## Graphical abstract

**Figure:**
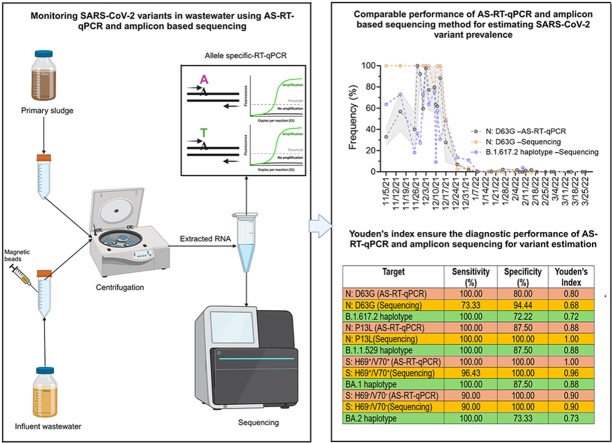

## 1 Introduction

The SARS-CoV-2 RNA genome is susceptible to mutations, resulting in the emergence of viral variants throughout the COVID-19 pandemic [1,2]. Many of the more prevalent variants have been linked to increased transmissibility, disease severity, reinfection, and reduced vaccine effectiveness [[3], [4], [5]]. These have been designated as variants of concern (VOCs), variants of interest (VOIs), and variants under monitoring (VUMs) [6]. Identifying and tracking them through clinical genomic surveillance has become critical to early and effective global public health responses [[7], [8]].

Clinical genomic surveillance of SARS-CoV-2 catalogues all mutations and variants with an individual case or patient granularity, but the method is expensive, time-consuming, and requires a sufficient number of clinical tests to ascertain the epidemiological metrics such as variant prevalence in a population [9,10]. Allele-specific reverse transcription quantitative PCR (AS-RT-qPCR, or its digital PCR counterpart) is another analytical tool that has been used to monitor SARS-CoV-2 mutations and variants in clinical specimens during the pandemic [11,12]. The clinical implementation of the method reports binary outcomes, either the presence or absence of a targeted mutation (allele). Although practically limited to monitoring one or two genetic loci for mutation, careful selection of sufficiently variant-specific mutations allows the method to be easily applied in a conventional SARS-CoV-2 diagnostic PCR testing facility and can rapidly report variant prevalence [13].

Despite the wide adoption of these tools during the pandemic, sequencing, or AS-RT-qPCR-based SARS-CoV-2 clinical surveillance may not accurately estimate the frequency of a viral variant circulating in asymptomatic, pre-symptomatic, and post-symptomatic cohorts (i.e., those unlikely to be tested) and its implementation at large scale (i.e., local, national and regional scale) is not sustainable [14]. The SARS-CoV-2, a respiratory virus, and its RNA shed in various bodily fluids, feces, and urine, which eventually enter sewer networks from infected individuals, including symptomatic, asymptomatic, and presymptomatic patients [[15], [16]]. This finding leads to the emergence of wastewater-based surveillance as a non-invasive method for tracking the presence and prevalence of the virus across populations. Later, wastewater-based genomic and AS-RT-qPCR-based surveillance have been rapidly developed and adopted by public health departments and agencies to monitor SARS-CoV-2 variants. The resulting data provide an aggregated, anonymous, and non-invasive measurement of the prevalence of circulating SARS-CoV-2 variants in the community [[17], [18], [19], [20]].

The power of wastewater-based surveillance of SARS-CoV-2 genetic variation is in its ability to cast a much wider net than its clinical counterpart, allowing the discovery of new and emerging or cryptic (i.e., not found in clinical genome repositories) mutations that might have clinical significance [21,22]. However, it is fundamentally different from its clinical counterpart, which derives epidemiological metrics such as variant prevalence from discrete counts (i.e., presence/absence enumerated from individual cases). Wastewater variant frequency represents continuous variables, convolved from multiple individuals with different fractional contributions and different states of genomic degradation and fragmentation. This makes it challenging to deconvolve and estimate the abundances of contributing SARS-CoV-2 haplotypes (i.e., the set of mutations that make up the viral genome) in the wastewater context. A reductionist solution to this problem is to track a single allele diagnostic for a given variant. Methods for both approaches have been successfully implemented (i.e., haplotype-based variant frequency estimation from sequencing, and single-allele variant frequency estimation from AS-RT-qPCR) in the wastewater context, providing valuable community incidence/prevalence data to public health [19,23,24]. However, monitoring of a single allele is prone to signal dropout due to differential fragmentation, degradation, or expression of the locus in different wastewater contexts, thus increasing the risk of false negative detection [25,26]. In addition, the re-emergence of a single allele with multiple variants could lead to inaccurate frequency estimations.

Two studies have shown that both AS-RT-qPCR and AS-RT-dPCR are more sensitive compared to sequencing-based methods for variant detection and quantification in wastewater [27,28]. Previous studies [29,30] also revealed that single-allele frequency estimations obtained by AS-RT-dPCR were strongly correlated with single-allele or haplotype frequency estimates derived from sequencing. However, the diagnostic performance (i.e., accuracy) of these wastewater-based methods with respect to the actual, real-world data of clinical surveillance remains unclear. The diagnostic performance of an assay is derived by enumerating true positive (TP), false positive (FP), true negative (TN), and false negative (FN) detections using a gold-standard test as a benchmark. Youden's index, which reports sensitivity and specificity in a single metric ranging from 0 to 1 and reflects test accuracy, is used widely to measure the performance of clinical diagnostic tests [[31], [32], [33], [34]]. A value of 1 indicates a perfect test with no false positive or negative detections, while 0 represents a test that detects equal proportions of false positives and false negatives, making it ineffective [35,36]. Assigning this index to test the presence or absence of SARS-CoV-2 variants in wastewater samples could help assay development teams in choosing the best allele or set of alleles with which to estimate variant frequencies.

In this study, four alleles associated with B.1.617.2 (Delta; N: D63G), B.1.1.529 (Omicron; N: P13L), and Omicron sub-lineages BA.1 (S: H69^+^/V70^+^) and BA.2 (S: H69^–^/V70^–^), were individually evaluated using AS-RT-qPCR and amplicon-based sequencing methods across periods of their emergence, dominance, and extinction. Single, variant diagnostic allele frequencies measured by AS-RT-qPCR were compared to both single-allele and haplotype frequencies measured by sequencing. Both AS-RT-qPCR and sequencing based on single-allele and haplotype frequencies were found to be comparable in describing the trajectory of variant prevalence. To quantitatively evaluate the accuracy of these methods in detecting variant frequency in wastewater, Youden's index was applied for each method using Ontario, Canada's clinical genomic surveillance data as the benchmark. Youden's index confirmed the accuracy of these methods for estimating the frequency of the SARS-CoV-2 variant in wastewater.

## 2 Materials and methods

### 2.1 Wastewaters sampling

Thirty-six parallel (post-grit influent and primary sludge) 24-h composite samples were collected from the City of Ottawa's Robert O. Pickard Environmental Center (ROPEC), Ontario, Canada using autosamplers (Hoskin Scientific, Burlington, Canada) between November 5, 2021 and April 12, 2022. On each sampling day, 500 mL influent and primary sludge samples were collected over 24 h. Wastewater samples were transferred to the laboratory in ice cooler packs and stored at 4 °C until analysis. The primary sludge samples were analyzed by AS-RT-qPCR within 24 h of sampling in Ottawa and influent samples were shipped to National Microbiology Laboratory (NML), Winnipeg, Canada using ice cooler packs for SARS-CoV-2 whole genome sequencing.

### 2.2 RNA enrichment and extraction

For AS-RT-qPCR, total RNA from primary sludge was extracted using the RNeasy Power Microbiome Kit (Qiagen, Germantown, USA) and a QIACube connect automated extraction platform as previously described [37]. Briefly, primary sludge samples were mixed thoroughly, 40 mL transferred to centrifuge tubes, and centrifuged for 45 min at 10,000 × *g* at 4 °C. The supernatant was discarded, and the pellet was further centrifuged at 10,000 × *g* for 5 min at 4 °C, and 250 ± 5 mg of the pellet was transferred to an RNase-free microfuge tube. The pellet was lysed with 650 μL PM1 buffer with β-mercaptoethanol (100:1 ratio) followed by the addition of Trizol LS reagent (ThermoFisher, Ottawa, Canada) before vortexing and centrifugation. Then, ∼1 mL of lysate was applied to columns placed in the QIACube connect to complete the process. On-column DNase treatment was performed as per the manufacturer's instructions. 100 μL of total RNA was eluted using nuclease-free water.

For amplicon sequencing, Nanotrap® Magnetic Virus Particles (Ceres Nanosciences, Manassas, USA) were used to enrich SARS-CoV-2 RNA from 50 mL of influent wastewater. Samples were left at room temperature for 10 min in 50 mL conical tubes to settle the larger particles, and 40 mL of supernatant was transferred to centrifuge tubes. Then, 600 μL of magnetic particles were added, and samples were rotated end-over-end at 100 rpm for 20 min at 20 °C, then centrifuged for 10 min at 4 °C at 8000 × *g*. The centrifuge tubes were placed on a magnetic rack (DynaMag-50, Invitrogen, Waltham, USA), and the supernatant was discarded by pipetting without disturbing the pellet. The pellet was resuspended in 140 μL of phosphate-buffered saline (pH 7.4) and 560 μL of Viral Lysis Buffer from QIAmp Viral RNA Mini Kit (Qiagen). The resulting suspension was transferred to a 2.0 mL microcentrifuge tube and placed on a magnetic rack (Invitrogen™ DynaMag™-2 magnet) for 10 min to remove the magnetic particles. The lysate was carefully collected by pipette, and total nucleic acid was extracted using QIAmp Viral RNA Mini Kit (Qiagen) and eluted in 68 μL of nuclease-free water. We measured A260/A280 (protein and phenol) and A260/A230 (phenol and guanidine) ratios using a Nanodrop for a couple of samples extracted via centrifugation and Nanotrap Magnetic Virus Particles. We found that the A260/A280 ratio ranged from 1.8 to 2.0, while the A260/A230 ratio was higher than 2.0, indicating high purity of the extracted RNA.

### 2.3 Allele-specific RT-qPCR

The presence of SARS-CoV-2 RNA in the samples was confirmed by singleplex RT-qPCR targeting N1 and N2 loci using TaqMan® Fast Virus 1-Step Master Mix (ThermoFisher) on a CFX Connect qPCR thermocycler (Bio-Rad, Hercules, Canada). Samples were analyzed in triplicates, with RNAse-free water serving as the non-template control, and quantified using a five-point gradient of the EDX SARS-CoV-2 standard curve (Exact Diagnostics, Texas, USA). The EDX standard is commonly used to quantify SARS-CoV-2 targets, including E, N, ORF1ab, RdRp, and S genes, with concentrations reaching up to 200 cp/μL. In contrast, AS-RT-qPCR was performed using previously published methods (Table S1) to quantify frequencies of the following alleles diagnostic for Delta and Omicron variants across the study period: 1) N: D63G (targeting Delta B.1.617.2 and its sub-lineages); 2) N: P13L (targeting Omicron B.1.1.529 and its sub-lineages); 3) S: H69^+^/V70^+^ (targeting Omicron B.1.1.529.1 alias BA.1) and 4) S: H69^–^/V70^–^(targeting Omicron B.1.1.529.2 alias BA.2). The AS-RT-qPCR reactions were considered positive when threshold cycle (Ct) < 40. The use of internal controls and their recovery efficiency was conducted by D'Aoust et al. [38] in our laboratory. Following the methodology outlined in that publication, the data presented in this study have not been corrected for recovery efficiency. Besides, the method's limit of detection, as well as the qPCR data processing, were performed as per the MIQE recommendations [39]. Additionally, sample runs were discarded if they did not meet the following requirements: i) standard curves are linear (R^2^ ≥ 0.95), ii) copies/well are found in the linear range of the standard curve, iii) standard deviation among triplicates <0.5, and iv) no positives in the no-template controls.

### 2.4 SARS-CoV-2 tiled amplicon sequencing

First, DNA from wastewater extracts RNA was removed using ezDNase Enzyme kit (ThermoFisher) and cDNA was synthesized using Superscript IV First-Strand Synthesis System kit (ThermoFisher) according to the previously described protocol [40]. Amplicons (∼400 bp) were generated from the cDNA using Artic V3 (samples collected between November 5 and 30, 2021) or V4.1 (samples collected between December 1, 2021 and April 12, 2022) protocols, and the size of the amplicons was confirmed through the 2100 Bioanalyzer system (Agilent Technologies, Mississauga, Canada). The amplicons were purified with 0.8× AMPure XP beads (Beckman Coulter) and quantified with a Qubit 4 fluorometer (ThermoFisher) using the dsDNA High Sensitivity Kit (ThermoFisher). The libraries were prepared using Nextera XT DNA Library Preparation Kit (Illumina, San Diego, USA), quantified, normalized, pooled, and diluted to 10 pM. The pooled 10 pM library was sequenced on a MiSeq platform (Illumina) using a PE flow cell with 300 bp paired end-run mode.

### 2.5 Mapping and variant calling

A customized “nf-core/viralrecon” bioinformatics pipeline was used to analyze the raw sequences [41]. Briefly, the quality of raw reads was assessed using FastQC [42], filtered using fastp [43] to remove adaptor sequences, ambiguous bases, low-quality reads (Phred score < 30), and small fragments (<50 nt). The filtered reads were then aligned to the SARS-CoV-2 reference sequence (NCBI Nucleotide accession MN908947.3) using Bowtie2 with default parameters [44]. The aligned reads were sorted using SAMtools [45], and consensus sequences were generated using iVar [46]. The consensus sequence was constructed using map reads with coverage of >5× and a Phred score of >30. Finally, variant calling was performed using iVar with a minimum frequency threshold (0.01), minimum Phred score (30), and minimum read depth (30).

### 2.6 Estimation of allele frequency using AS-RT-qPCR

The frequency of the variant allele in wastewaters was calculated as a fraction of the sum of variant and reference allele RNA copies determined using allele-specific forward primers (D63G or P13L assays) or probe (H69^+^/V70^+^ assay) against a reference standard curve as previously described [19]. More detailed information is found in Table S1.

### 2.7 Haplotype construction of SARS-CoV-2 variants from wastewater

To construct the haplotype of each variant, mutations specific to each lineage were first selected from public repositories, including the GISAID database [47], CovSPECTRUM [48], and Pango Lineages [49]. Mutations shared between lineages were removed to reduce overestimation or false positive detections. A mutation was defined as specific if it was found predominately in ≥90% of the clinical genomes assigned to the lineage and absent or found in ≤10% of clinical genomes assigned to all other lineages. The average frequency across all of the specific mutations that defined a lineage (haplotype) was defined as the haplotype frequency.

### 2.8 Diagnostic performance assessment–Youden's index

The diagnostic performance of AS-RT-qPCR and amplicon-based sequencing methods for single allele and haplotype were evaluated based on Youden's index as follows [35,36].

Youden's index = (Sensitivity + Specificity) – 1

Sensitivity = *TP*/(*TP* + *FN*)

Specificity = *TN*/(*TN* + *FP*)

Where *TP* (true positive) was defined as the number of events with both a positive detection of the variant in wastewater samples and ≥5% of clinical genomes, whereas *TN* (true negative) was the number of events with both negative detection in wastewater and <5% of clinical genomes sequenced in Ontario in the period assigned to the variant in question [50]. On the contrary, *FP* (false positive) was the number of events with variant detection in wastewater but <5% of clinical genomes assigned, and *FN* (false negative) was the number of non-detection events in wastewater with ≥5% of clinical genomes assigned. Table S2 summarizes these events.

### 2.9 Statistical analysis

The Wilcoxon matched-pairs signed-rank test was used to test for statistically significant differences (*p* < 0.05) between AS-RT-qPCR and the amplicon-based sequencing method. Spearman rank correlation analysis was used to compute the relation of the single allele and haplotype frequency of each variant. The statistical analysis and graphs were generated with GraphPad Prism 10.2.1 (La Jolla, California, USA).

## 3 Results and discussion

### 3.1 Longitudinal comparison of AS-RT-qPCR and amplicon-based sequencing in wastewaters

Primary sludge and post-grit influent samples collected on the same day between November 5, 2021 and April 12, 2022, were subjected to analysis using AS-RT-qPCR and amplicon-based sequencing. This period of time saw distinct phases of the pandemic characterized by the replacement of the endemic Delta sub-lineages with those of Omicron in the sampled population. To compare AS-RT-qPCR with amplicon sequencing for estimating SARS-CoV-2 variant frequency, alleles were chosen based on the contemporaneously unique haplotype-defining mutations derived from clinical genomic surveillance (PANGO) as described in Materials and Methods. For sequencing, we chose unique alleles associated with B.1.617.2 (parental lineage and its sub-lineages), B.1.1.529 (parental lineage and its sub-lineages), B.1.1.529.1 (alias BA.1), and B.1.1.529.2 (alias BA.2). For AS-RT-qPCR, diagnostic alleles for Delta and its sub-lineages (N: D63G), Omicron and its sub-lineages (N: P13L), BA.1 (S: H69^+^/V70^+^), and BA.2 (S: H69^–^/V70^–^) were chosen based on contemporaneous prevalence in clinical genomic sequences. For each sampling date, variant frequency was estimated through a single, diagnostic allele by AS-RT-qPCR, the same allele by sequencing, and the haplotype by sequencing. We computed Spearman's correlation between the two sequencing-based estimates for each variant to determine if single-allele estimates reflected the computed average across the alleles making up the haplotype. This revealed statistically significant (*p* < 0.05) positive correlations (r = 0.860 to 0.903) for each variant (Fig. S1). Later we evaluated the performance of these three methods (i.e., AS-RT-qPCR, single-allele sequencing, and haplotype sequencing) in estimating the prevalence of variants in wastewater associated with the period of waning Delta (B.1.617.2) prevalence and the concomitant emergence of Omicron (B.1.1.529) in December 2021; and the replacement of the dominant BA.1 Omicron variant with the BA.2 sub-lineage in April 2022.

**Fig. 1:**
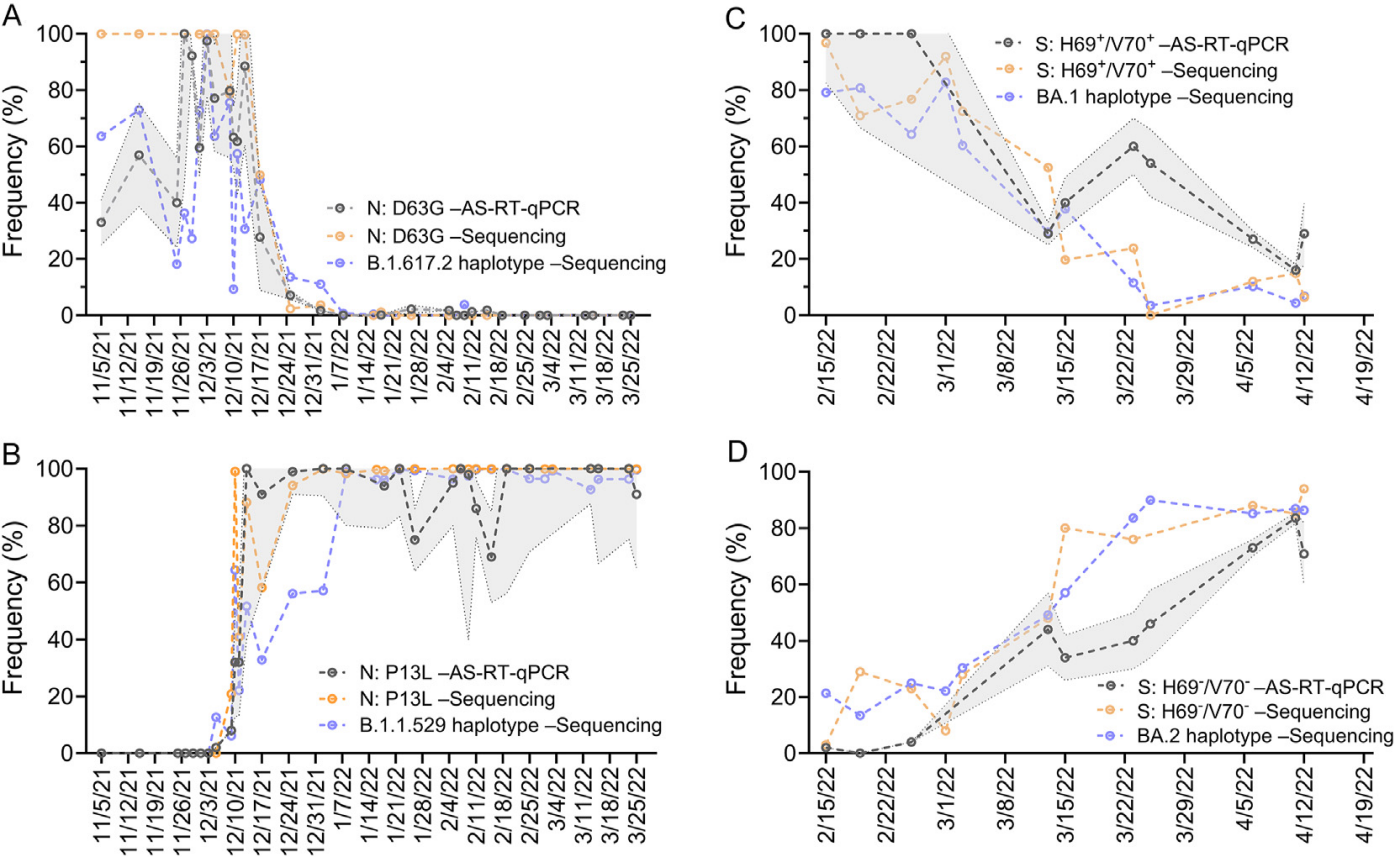
Comparison of AS-RT-qPCR and amplicon-based sequencing methods for estimating SARS-CoV-2 variants frequency in wastewaters; A) N: D63G and B.1.617.2 haplotype, B) N: P13L and B.1.1.529 haplotype, C) S: H69^+^/V70^+^ and BA.1 haplotype, and D) S: H69^–^/V70^–^ and BA.2 haplotype. Shadow area with AS-RT-qPCR represents the standard deviation of estimation of the targeted allele of a specific variant.

**Fig. 2:**
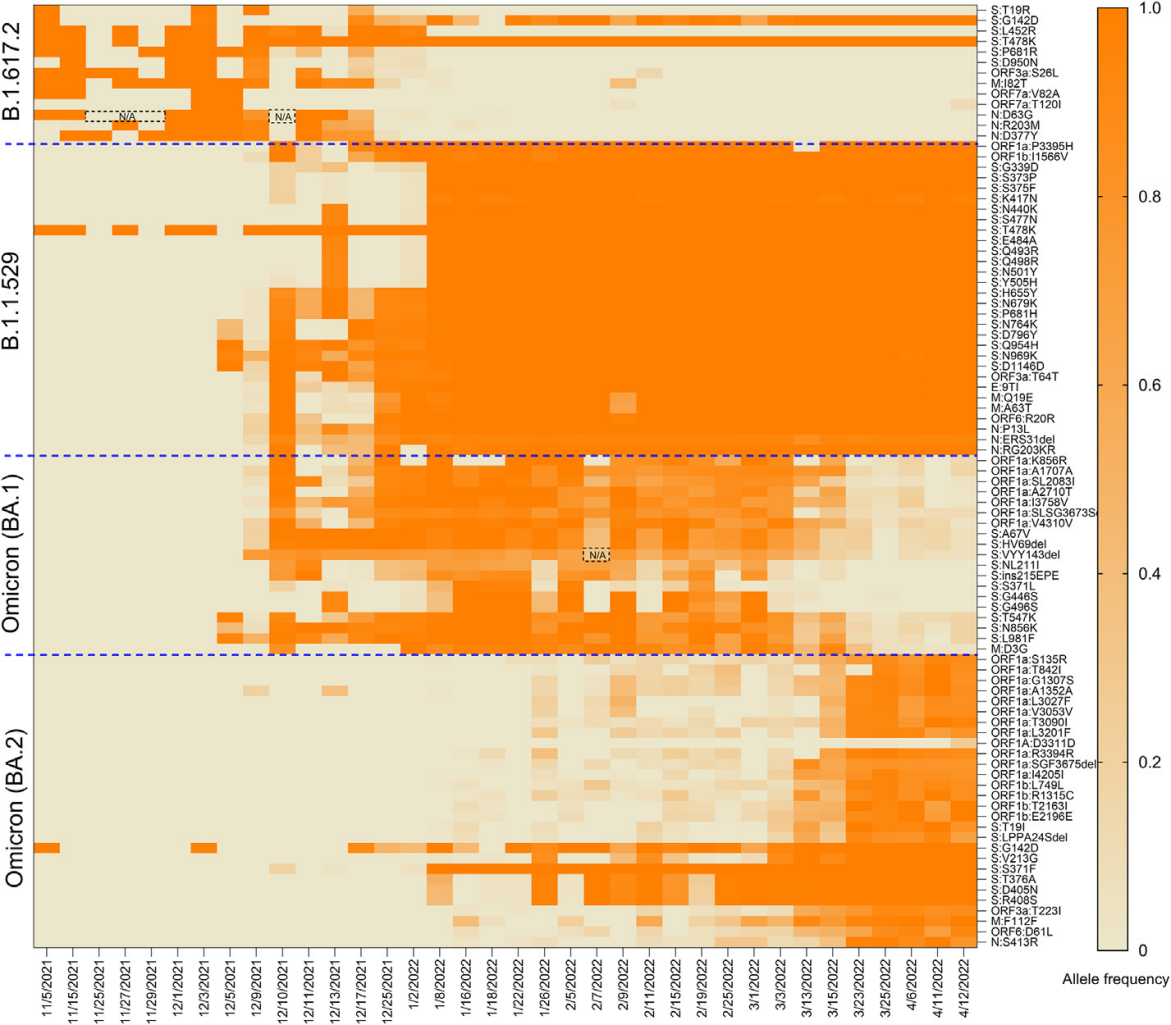
Allele frequency of variant-defining mutation of four different Pango lineages (B.1.617.2, B.1.1.529, BA.1, and BA.2) in wastewater from November 5, 2021 to April 12, 2022. The heat map represents the changes in the frequency of allele during the waning of Delta with the waxing of Omicron, as well as the waning of BA.1 with the waxing of BA.2. The missing data points of a single allele due to insufficient sequencing depth for mutation calling during the dominant period of the corresponding variant is indicated on the heatmap as N/A with black dash points.

In the first period, N: D63G frequency assessed by AS-RT-qPCR and B.1.617.2 (Delta) haplotype frequency derived by sequencing were on average 50% and 75% in November to early December 2021 samples, respectively, whereas N: D63G frequency measured by sequencing was 100% (Fig. 1A). The inconsistency of N: D63G allele frequency between AS-RT-qPCR and amplicon sequencing exhibited more sensitive detection of N: D63G in the sequencing-based method. In contrast, the variability of single allele (N: D63G) and B.1.617.2 haplotype was due to the numerous missing data points of haplotype-defining alleles because of insufficient sequencing depth (<30×) (Fig. 2). In the middle of December 2021, Delta was rapidly replaced by Omicron. The frequency estimates derived by AS-RT-qPCR or sequencing (haplotype or single-allele) for Delta dropped precipitously in the third week of December 2021. This observation was concomitant with an equally rapid increase in Omicron frequency estimates using all three methods (Fig. 1B). This pattern of variant prevalence was consistent with that derived from clinical genomic surveillance in Canada during this period [48]. The last days of Delta were characterized by very low overall SARS-CoV-2 signal in wastewater, and this was reflected in the lower frequencies of the Delta variant using any of the methods.

The Omicron-specific N: P13L allele was first detected in primary sludge using AS-RT-qPCR on December 5, 2021 (2% of total signal), while the same allele was not detectable on that date by sequencing from influent and was instead first detected on December 9, 2021, sample at a frequency of 20%, close to the estimate by AS-RT-qPCR on that day (Fig. 1B). The AS-RT-qPCR estimates were derived from RNA extracted from primary sludge, which we and others [[38], [51]] have previously been shown to have higher SARS-CoV-2 RNA concentrations, which may be the reason for the early detection of the N: P13L allele. Importantly, the trajectory of measured N: P13L or B.1.1.529 haplotype frequencies was consistent with that derived from clinical genomic surveillance in Canada [48]. The B.1.1.529 haplotype frequency took longer to reach its peak than N: P13L due to the lower frequency of certain alleles (S: N440K, S: S477N, S: E484A, S: Q493R, S: Q498R, M: Q19E, M: A63T) (Fig. 2).

We next assessed any differences in the three methods during the late winter to early spring period of 2022 when an Omicron BA.2 epi-wave was displaced by the endemic BA.1 [47,49]. Here, allele frequencies of S: H69^+^/V70^+^ were monitored by AS-RT-qPCR or by sequencing. This allele distinguishes BA.1 from B.1.1.529 or BA.2, the latter two harboring a deletion at this locus. At the beginning of the monitoring period, S: H69^+^/V70^+^ frequencies were similar (*p* > 0.05) between AS-RT-qPCR and sequencing-based methods, but two sample dates near the end of March 2022 showed a larger deviation between AS-RT-qPCR and two sequencing-based methods (Fig. 1C). A similar deviation was also observed between AS-RT-qPCR (S: H69^–^/V70^–^) and BA.2 haplotype frequency estimates (Fig. 1D). The S: H69^–^/V70^–^ allele was realistically absent in BA.2, and during the initial stage of BA.2 epi-wave, there was no locus being monitored by AS-RT-qPCR. As epi-wave BA.2 replaced BA.1, it was presumed that the prevalence of S: H69^–^/V70^–^ could be estimated through (100% – frequency of S: H69^+^/V70^+^) since the deleted “ATACATG” nucleotides in BA.1 returned with BA.2. Later, we found the similar (*p* > 0.05) trajectory of S: H69^–^/V70^–^ and BA.2 haplotype frequency in wastewater in all three methods (Fig. 1D). Overall, these findings revealed comparable trends of SARS-CoV-2 variant frequency across the methods employed, although certain differences were observed on particular days. These findings are consistent with earlier studies, which demonstrated no discernible differences between AS-RT-qPCR or AS-RT-dPCR and sequencing-based methods for estimating SARS-CoV-2 mutations or variants prevalence in wastewater [28,52].

### 3.2 Diagnostic performance of AS-RT-qPCR and amplicon-based sequencing in wastewaters

The accuracy of AS-RT-qPCR and amplicon-based sequencing methods was evaluated for each variant by quantifying diagnostic sensitivity and specificity across the sampling periods using the presence/absence of the variant in clinical samples as the benchmark test. In this respect, we employed Youden's index, where an index value of 1 indicates a perfect test (i.e., as sensitive and specific as clinical genomic surveillance). Such parameterization of the method performance can help establish optimal thresholds for the inclusion/exclusion of alleles used to measure the frequency (i.e., prevalence) of SARS-CoV-2 variants in communities using wastewater-based genomic surveillance. Here, we evaluated Youden's index of targeted alleles accessed by AS-RT-qPCR, haplotype-defining mutations of each variant, and haplotype of each variant. Results showed that Youden's indices of single allele frequency by AS-RT-qPCR (N: D63G, N: P13L, S: H69^+^/V70^+^ and S: H69^–^/V70^–^) were 0.80, 0.88, 0.96 and 0.90, while those for the same targets using sequencing were 0.68, 1.00, 0.96 and 0.90, respectively (Table 1). Youden's indices of B.1.617.2, B.1.1.529, BA.1, and BA.2 haplotype-based detections were 0.72, 0.88, 0.88, and 0.73, respectively. Although Youden's indices across AS-RT-qPCR, single allele frequency sequencing and haplotype frequency sequencing were comparable (*p* > 0.05), the lower index of N: D63G in the single-allele sequencing method was driven by the exclusion of four data points (November 25, 27, 29, and December 10, 2021) due to insufficient sequencing depth (<30×) that precluded mutation detection at this locus during the dominant period of Delta variant. The exclusion of data points due to insufficient sequencing depth was also observed in the other locus (Fig. 2). Sequencing depth is a pivotal determinant of accurate SARS-CoV-2 mutation detection, as low read depth reduces the likelihood of accurately identifying mutations, making it difficult to distinguish true alleles from sequencing errors [28,53]. This can result in incomplete or biased allele frequency estimates, which eventually influence Youden's index by lowering the sensitivity of the alleles. On the contrary, reduced specificity affects the Youden's index for AS-RT-qPCR, which was driven by the lower proportion (∼2%) of N: D63G detection in wastewater for a few days (January 26, February 5, 11 and 15, 2022) during a period when no Delta cases were detected in the Ontario clinical sequences (Fig. 1A). This could be due to fewer cases (so less likely to be detected and sequenced through clinical surveillance) and/or persistent infection and shedding from prior incident cases. Detection of B.1.617.2 haplotype (i.e., ORF3a: S26L, M: I82T, ORF7a: T120I, N: R203M) in this period is consistent with these possibilities (Fig. 2). Similarly, the early detection of certain alleles in wastewater relative to clinical surveillance affected the specificity of B.1.1.529, BA.1, and BA.2 haplotypes, resulting in decreased Youden's indices.

**Table 1:**
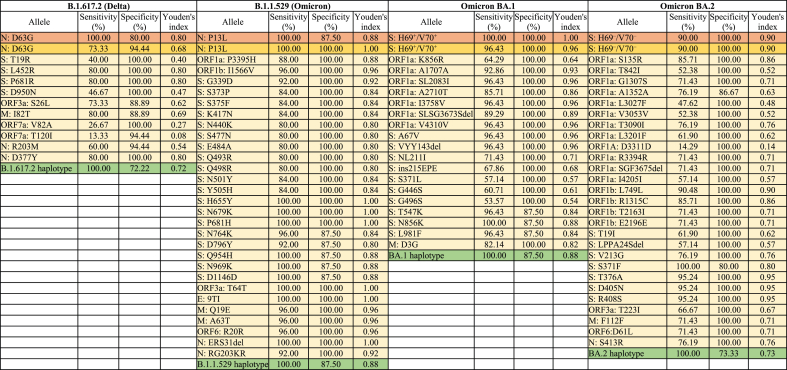
Youden's indices of alleles uniquely associated with B.1.617.2 (Delta), B.1.1.529 (Omicron), BA.1 (Omicron B.1.1.529.1), BA.2 (Omicron B.1.1.529.2), the haplotype of each variant, as well as N: D63G, N: P13L, S: H69^+^/V70^+^ and S: H69^*–*^/V70^*–*^ using AS-RT-qPCR in wastewaters.

The orange color cells indicate the Youden's index for alleles evaluated with AS-RT-qPCR, yellow represents the Youden's index for the same alleles in sequencing, light yellow denotes the Youden's index uniquely associated with each variant evaluated by sequencing, and green represents the Youden's index of the haplotype of each variant.

Although the Youden's index threshold for a perfect diagnostic test is 1, the highest Youden's indices for haplotype-defining mutations of B.1.617.2, B.1.1.529, BA.1, and BA.2 variants in wastewater context were 0.80, 1.00, 0.96, and 0.95, respectively (Table 1). The highest Youden's index for each variant signified almost an ideal target allele, and the alleles we targeted using AS-RT-qPCR or sequencing-based methods aligned perfectly with the Youden's index threshold (i.e., 1). This indicated the accuracy of wastewater-based genomic surveillance using AS-RT-qPCR or sequencing-based methods against the clinical genomic surveillance for estimating SARS-CoV-2 variants prevalence in a population. However, suitable allele selection can be challenging as numerous alleles contain similar Youden's indices, and alleles can be influenced by sufficient sequencing depth in sequencing-based methods. Thereby, more than one allele can be separately monitored to overcome the read depth limitations of specific mutations. Youden's index of haplotype-based estimation was lower than the threshold of each variant, as monitoring haplotypes has the potential of false positive and negative detection due to the presence of certain alleles during the waning or waxing of each variant. These highlight a known issue: curating accurate haplotypes for Pango lineages is a challenging, and ongoing task in the face of rapidly emerging variants and diminishing SARS-CoV-2 clinical genomic surveillance.

### 3.3 Practical considerations of wastewater AS-RT-qPCR and amplicon-based sequencing implementation

AS-RT-qPCR is a rapid and inexpensive method for estimating SARS-CoV-2 variants frequency in wastewater compared to amplicon-based sequencing methods. Starting with extracted RNA, the turnaround time of AS-RT-qPCR can be as little as 2 h, including data analysis with an estimated reagent cost of ∼USD$15 per sample. In contrast, amplicon-based SARS-CoV-2 genome sequencing takes about 34 h in five consecutive steps, including cDNA synthesis, amplicon generation, library preparation, sequencing, and data analysis. The reagents cost of the amplicon-based sequencing method is ∼USD$100/sample (up to 24 samples with a MiSeq sequencing run). Despite the longer turnaround time and higher reagent costs, the sequencing-based method can identify the number and relative abundance of numerous mutations and variants present in wastewater without prior knowledge of the mutations. In contrast, AS-RT-qPCR requires prior knowledge of a diagnostic mutation, and this precludes its use as a method to discover emerging variants unless the new variant has undergone a reversion at a locus currently being monitored by AS-RT-qPCR (e.g., S: H69^+^/V70^+^). Significant time and expertise are needed to design and implement a new AS-RT-qPCR assay. However, the time-to-delivery of new assays has been significantly reduced during the pandemic with a better understanding of what works well and re-using existing assays as new variants replace old ones.

The diagnostic performance evaluated using Youden's index of AS-RT-qPCR and amplicon-based sequencing methods demonstrated that all three methods can be reliably used for estimating SARS-CoV-2 variants in wastewater, with some precautions. The Youden's index of AS-RT-qPCR was affected by reduced specificity due to low frequency detected during a period when no clinical cases were detected. On the contrary, the single-allele sequencing method was susceptible to a lower Youden's index (reduced sensitivity) when there was insufficient depth to confidently detect the mutation. Thus, monitoring more than one allele separately using both methods can help to overcome the sensitivity and specificity limitations imposed by lower frequency and read depth and results in the increased accuracy of AS-RT-qPCR and sequencing-based methods for single allele estimation. The haplotype method, which should have high specificity, exhibited false positive detection during variant transition periods when the emerging and endemic variants shared alleles. When multiple variants share common alleles (genetic markers), it becomes more challenging to definitively assign detected alleles to a specific variant. This overlap can reduce both specificity and sensitivity, potentially resulting in an underestimation of the true positive rate or an overestimation of the false positive rate, thus complicating accurate variant identification. Thus, the accuracy of haplotype estimation requires careful haplotype curation. Accurate haplotypes can be assigned retrospectively. However, prospective curation is challenging given the potential lag between the appearance of signal in wastewater and the presence of the curated genomes in clinical surveillance databases.

In general, AS-RT-qPCR offers rapid, targeted detection of specific viral variants or alleles, while sequencing provides comprehensive genomic insights, enabling the identification of novel mutations and characterization of entire viral genomes. Though both methods exhibit similar diagnostic capabilities, their implementation faces challenges, particularly in low and lower-middle-income countries with limited resources. In rural and resource-limited settings, AS-RT-qPCR can support long-term surveillance and pandemic preparedness, where understanding the trajectory of pathogen mutations is crucial for public health response. However, AS-RT-qPCR alone may fall short when emerging variants or unknown mutations arise. In this context, sequencing facilitates the discovery of novel variants, provides transmission insights, and enables retrospective analyses of variant evolution. Integrating AS-RT-qPCR and sequencing into routine wastewater surveillance holds transformative potential for early detection and control of infectious diseases. The combined use of AS-RT-qPCR's rapid detection with sequencing's detailed genomic analysis strengthens our capacity to monitor pathogen evolution and spread, contributing to more effective public health responses and future pandemic preparedness.

## 4 Conclusion

In conclusion, our results show the similar performance of AS-RT-qPCR and sequencing-based methods to estimate SARS-CoV-2 variants frequency in wastewater using a single diagnostic allele or haplotype as a proxy of variant prevalence in a community. Later, we evaluated diagnostic performance by employing Youden's index to quantitate the accuracy of each method against the clinical genome surveillance and found that when an emerging SARS-CoV-2 variant signal is very low, the diagnostic specificity of monitoring a single-allele by AS-RT-qPCR is reduced (or clinical genomic surveillance sampling rate is insufficient). Monitoring the same allele via sequencing exhibited reduced sensitivity due to insufficient sequencing depth at the targeted locus. Youden's index derived from haplotype detections of each variant was influenced by the presence of shared alleles, highlighting the need for careful haplotype curation to accurately estimate frequencies. Therefore, the use of more than one allele using AS-RT-qPCR and amplicon-based sequencing methods is recommended during periods of low signal and low cases to overcome the sensitivity limitations of each method. Our study recommends that any one of the methods evaluated here would provide public health stakeholders with trajectories of SARS-CoV-2 variants in their community. However, their integrations provide a comprehensive picture of the circulating variants and assist public health decision-making.

## CRediT authorship contribution statement

**Md Pervez Kabir:** Writing – review & editing, Writing – original draft, Visualization, Software, Methodology, Investigation, Formal analysis, Data curation, Conceptualization. **Élisabeth Mercier:** Writing – review & editing, Formal analysis. **Walaa Eid:** Writing – review & editing, Investigation. **Julio Plaza-Diaz:** Writing – review & editing, Investigation. **Patrick M. D'Aoust:** Writing – review & editing, Investigation. **Chrystal Landgraff:** Writing – review & editing, Investigation. **Lawrence Goodridge:** Writing – review & editing. **Opeyemi U. Lawal:** Writing – review & editing. **Shen Wan:** Writing – review & editing, Investigation. **Nada Hegazy:** Writing – review & editing. **Tram Nguyen:** Writing – review & editing. **Chandler Wong:** Writing – review & editing. **Ocean Thakali:** Writing – review & editing. **Lakshmi Pisharody:** Writing – review & editing. **Sean Stephenson:** Writing – review & editing. **Tyson E. Graber:** Writing – review & editing, Validation, Supervision. **Robert Delatolla:** Writing – review & editing, Validation, Supervision, Methodology, Funding acquisition.

## Declaration of competing interest

The authors declare that no known competing financial interests or personal relationships influenced the work reported in this manuscript.
