# Supplementary material for: Diagnostic performance of allele-specific RT-qPCR and genomic sequencing in wastewater-based surveillance of SARS-CoV-2
Source: Eco Environ Health. 2025 Jan 21;4(1):100135. doi: 10.1016/j.eehl.2025.100135 (PMC11992540; doi:10.1016/j.eehl.2025.100135)
Supplement: Multimedia component 1 [file mmc1.docx]

**Diagnostic performance of allele-specific RT-qPCR and genomic sequencing in wastewater-based surveillance of SARS-CoV-2**

Md Pervez Kabir^a^, Élisabeth Mercier^a^, Walaa Eid^b^, Julio Plaza-Diaz^b^, Patrick M. D’Aoust^a^, Chrystal Landgraff^c^, Lawrence Goodridge^d^, Opeyemi U. Lawal^d^, Shen Wan^a^, Nada Hegazy^a^, Tram Nguyen^a^, Chandler Wong^a^, Ocean Thakali^a^, Lakshmi Pisharody^a^, Sean Stephenson^b^, Tyson E. Graber^b^, and Robert Delatolla^a*^

^a^ Department of Civil Engineering, University of Ottawa, Ottawa, Ontario, Canada

^b^ Children's Hospital of Eastern Ontario Research Institute, Ottawa, Ontario, Canada

^c^ Division of Enteric Diseases, National Microbiology Laboratory, Public Health Agency of Canada, Winnipeg, Manitoba, Canada

^d^ Canadian Research Institute for Food Safety, Department of Food Science, University of Guelph, Guelph, Ontario, Canada

Corresponding author:
**Dr. Robert Delatolla**
Work E-mail: [Robert.Delatolla@uOttawa.ca](mailto:Robert.Delatolla@uOttawa.ca)

**Supplementary Material**

Table S1 Oligonucleotide sequences and AS-RT-qPCR reaction specifications of the targeted alleles associated with SARS-CoV-2 variants.

| Assay | Primer/probe | Sequence | PCR reaction specifications | References |
| --- | --- | --- | --- | --- |
| CDC N1 | 2019-nCoV_N1-F_Primer | GAC CCC AAA ATC AGC GAA AT | 50 ℃ for 5 min RT, 95 ℃ for 20 sec, and 45 cycles of 95 ºC for 3 sec, 60 °C for 30 sec | (CDC, 2020) |
|  | 2019-nCoV_N1-R_Primer | TCT GGT TAC TGC CAG TTG AAT CTG |  |  |
|  | 2019-nCoV_N1-Probe | **6-FAM**-ACC CCG CAT/**ZEN**/ TAC GTT TGG TGG ACC-**IBFQ** |  |  |
| CDC N2 | 2019-nCoV_N2-F_Primer | TTA CAA ACA TTG GCC GCA AA |  |  |
|  | 2019-nCoV_N2-R_Primer | GCG CGA CAT TCC GAA GAA |  |  |
|  | 2019-nCoV_N2-Probe | **6-FAM**-ACA ATT TGC/**ZEN**/CCC CAG CGC TTC AG-**IBFQ** |  |  |
| D63G (MT) | Forward primer | TCA CTC AAC ATG GCA AGA AAG G | 50 ℃ for 5 min RT, 95 ℃ for 20 sec, and 44 cycles of 95 ºC for 3 sec, 55 °C for 45 sec | (D’Aoust et al., 2022) |
|  | Reverse primer | GGT AGT AGC CAA TTT GGT CAT CT |  |  |
|  | Probe | **6-FAM**-CCT TAA ATT CCC TCG ATG ACA AGG CG-**MGB** |  |  |
| N63 (WT) | Forward primer | CTC ACT CAA CAT GGC AAG AAA G |  |  |
| P13L (MT) | Forward primer | CCA AAA TCA GCG AAA TGA ACC | 50 ℃ for 5 min RT, 95 ℃ for 20 sec, and 45 cycles of 95 ºC for 3 sec, 60 °C for 30 sec |  |
|  | Reverse primer | TCT GGT TAC TGC CAG TTG AAT CTG |  |  |
|  | N1 probe (version 2) | **6-FAM**-CCG CAT TAC GTT TGG TGG ACC C-**MGB** |  |  |
| H69^+^/V70^+^ (MT) | Forward primer | CATTCAACTCAGGACTTGTTCTTACC | 50 ℃ for 15 min RT, 95 ℃ for 2 min, 40 cycles of 95 ℃ for 5s and 60 ℃ for 30s | (Peterson et al., 2022) |
|  | Reverse primer | GGTAGGACAGGGTTATCAAACCTC |  |  |
|  | Probe | **FAM-**TCCATGCTATCTCTG-**MGB** |  |  |
| H69^+^/V70^+^ (WT) | Forward primer | CATTCAACTCAGGACTTGTTCTTACC |  |  |
|  | Reverse primer | GGTAGGACAGGGTTATCAAACCTC |  |  |
|  | Probe | **FAM**-ATGCTATACATGTCTCTG-**MGB** |  |  |

Table S2 The details including the dates of TP, FP, TN, and FN determination in wastewater against the Ontario clinical genomic surveillance for alleles associated with each variant, haplotype of each variant, and single allele of AS-RT-qPCR.

| **Lineage** | **Alleles and haplotype** | **TP detection dates** | **Number of TP detected** | **FN detection dates** | **Number of FN detected** | **TN detection dates** | **Number of TN detected** | **FP detection dates** | **Number of FP detected** |
| --- | --- | --- | --- | --- | --- | --- | --- | --- | --- |
| Delta (B.1.617.2) | S: T19R | 05-11-2021 to 02-01-2022 | 6 | 05-11-2021 to 02-01-2022 | 9 | 03-01-2022 to 25-03-2022 | 18 | 03-01-2022 to 25-03-2022 | 0 |
|  | S: L452R |  | 12 |  | 3 |  | 18 |  | 0 |
|  | S: P681R |  | 12 |  | 3 |  | 18 |  | 0 |
|  | S: D950N |  | 7 |  | 8 |  | 18 |  | 0 |
|  | ORF3a: S26L |  | 11 |  | 4 |  | 16 |  | 2 |
|  | M: I82T |  | 12 |  | 3 |  | 16 |  | 2 |
|  | ORF7a: V82A |  | 4 |  | 11 |  | 18 |  | 0 |
|  | ORF7a: T120I |  | 2 |  | 13 |  | 17 |  | 1 |
|  | N: D63G |  | 11 |  | 4 |  | 17 |  | 1 |
|  | N: R203M |  | 9 |  | 6 |  | 17 |  | 1 |
|  | N: D377Y |  | 12 |  | 3 |  | 18 |  | 0 |
|  | B.1.617.2 haplotype |  | 15 |  | 0 |  | 13 |  | 5 |
|  | N: D63G (AS-RT-qPCR) |  | 15 |  | 0 |  | 14 |  | 4 |
| Omicron (B.1.1.529) | ORF1a: P3395H | 09-12-2021 to 25-03-2022 | 22 | 09-12-2021 to 25-03-2022 | 3 | 05-11-2021 to 08-12-2022 | 8 | 05-11-2021 to 08-12-2022 | 0 |
|  | ORF1b: I1566V |  | 24 |  | 1 |  | 8 |  | 0 |
|  | S: G339D |  | 23 |  | 2 |  | 8 |  | 0 |
|  | S: S373P |  | 21 |  | 4 |  | 8 |  | 0 |
|  | S: S375F |  | 21 |  | 4 |  | 8 |  | 0 |
|  | S: K417N |  | 21 |  | 4 |  | 8 |  | 0 |
|  | S: N440K |  | 20 |  | 5 |  | 8 |  | 0 |
|  | S: S477N |  | 20 |  | 5 |  | 8 |  | 0 |
|  | S: E484A |  | 20 |  | 5 |  | 8 |  | 0 |
|  | S: Q493R |  | 20 |  | 5 |  | 8 |  | 0 |
|  | S: Q498R |  | 20 |  | 5 |  | 8 |  | 0 |
|  | S: N501Y |  | 21 |  | 4 |  | 8 |  | 0 |
|  | S: Y505H |  | 21 |  | 4 |  | 8 |  | 0 |
|  | S: H655Y |  | 25 |  | 0 |  | 8 |  | 0 |
|  | S: N679K |  | 25 |  | 0 |  | 8 |  | 0 |
|  | S: P681H |  | 25 |  | 0 |  | 8 |  | 0 |
|  | S: N764K |  | 24 |  | 1 |  | 7 |  | 1 |
|  | S: D796Y |  | 23 |  | 2 |  | 7 |  | 1 |
|  | S: Q954H |  | 25 |  | 0 |  | 7 |  | 1 |
|  | S: N969K |  | 25 |  | 0 |  | 7 |  | 1 |
|  | S: D1146D |  | 25 |  | 0 |  | 7 |  | 1 |
|  | ORF3a: T64T |  | 25 |  | 0 |  | 8 |  | 0 |
|  | E:9TI |  | 25 |  | 0 |  | 8 |  | 0 |
|  | M: Q19E |  | 24 |  | 1 |  | 8 |  | 0 |
|  | M: A63T |  | 24 |  | 1 |  | 8 |  | 0 |
|  | ORF6:R20R |  | 24 |  | 1 |  | 8 |  | 0 |
|  | NC |  | 11 |  | 14 |  | 8 |  | 0 |
|  | N: P13L |  | 25 |  | 0 |  | 8 |  | 0 |
|  | N: ERS31del |  | 25 |  | 0 |  | 8 |  | 0 |
|  | N: RG203KR |  | 23 |  | 2 |  | 8 |  | 0 |
|  | B.1.1.529 haplotype |  | 25 |  | 0 |  | 7 |  | 1 |
|  | N: P13L (AS-RT-qPCR) |  | 25 |  | 0 |  | 7 |  | 1 |
| Omicron BA.1 | ORF1a: K856R | 09-12-2021 to 12-04-2022 | 18 | 09-12-2021 to 12-04-2022 | 10 | 05-11-2021 to 08-12-2022 | 8 | 05-11-2021 to 08-12-2022 | 0 |
|  | ORF1a: A1707A |  | 26 |  | 2 |  | 8 |  | 0 |
|  | ORF1a:SL2083I |  | 27 |  | 1 |  | 8 |  | 0 |
|  | ORF1a: A2710T |  | 24 |  | 4 |  | 8 |  | 0 |
|  | ORF1a: I3758V |  | 27 |  | 1 |  | 8 |  | 0 |
|  | ORF1a: SLSG3673Sdel |  | 25 |  | 3 |  | 8 |  | 0 |
|  | ORF1a: V4310V |  | 27 |  | 1 |  | 8 |  | 0 |
|  | S: A67V |  | 27 |  | 1 |  | 8 |  | 0 |
|  | S: HV69del |  | 27 |  | 1 |  | 8 |  | 0 |
|  | S: VYY143del |  | 27 |  | 1 |  | 8 |  | 0 |
|  | S: NL211I |  | 20 |  | 8 |  | 8 |  | 0 |
|  | S:ins215EPE |  | 19 |  | 9 |  | 8 |  | 0 |
|  | S: S371L |  | 16 |  | 12 |  | 8 |  | 0 |
|  | S: G446S |  | 17 |  | 11 |  | 8 |  | 0 |
|  | S: G496S |  | 15 |  | 13 |  | 8 |  | 0 |
|  | S: T547K |  | 27 |  | 1 |  | 7 |  | 1 |
|  | S: N856K |  | 28 |  | 0 |  | 7 |  | 1 |
|  | S: L981F |  | 27 |  | 1 |  | 7 |  | 1 |
|  | M: D3G |  | 23 |  | 5 |  | 8 |  | 0 |
|  | Omicron BA.1 haplotype |  | 28 |  | 0 |  | 7 |  | 1 |
|  | S: H69^+^/V70^+^ (AS-RT-qPCR) | 15-02-2022 to 12-04-2022 | 10 | 15-02-2022 to 12-04-2022 | 0 |  | N/A |  | N/A |
| Omicron BA.2 | ORF1a: S135R | 08-01-2022 to 12-04-2022 | 18 | 08-01-2022 to 12-04-2022 | 3 | 05-11-2021 to 07-01-2022 | 15 | 05-11-2021 to 07-01-2022 | 0 |
|  | ORF1a: T842I |  | 11 |  | 10 |  | 15 |  | 0 |
|  | ORF1a: G1307S |  | 15 |  | 6 |  | 15 |  | 0 |
|  | ORF1a: A1352A |  | 16 |  | 5 |  | 13 |  | 2 |
|  | ORF1a: L3027F |  | 10 |  | 11 |  | 15 |  | 0 |
|  | ORF1a: V3053V |  | 11 |  | 10 |  | 15 |  | 0 |
|  | ORF1a: T3090I |  | 16 |  | 5 |  | 15 |  | 0 |
|  | ORF1a: L3201F |  | 13 |  | 8 |  | 15 |  | 0 |
|  | ORF1A:D3311D |  | 3 |  | 18 |  | 15 |  | 0 |
|  | ORF1a: R3394R |  | 15 |  | 6 |  | 15 |  | 0 |
|  | ORF1a: SGF3675del |  | 15 |  | 6 |  | 15 |  | 0 |
|  | ORF1a: I4205I |  | 12 |  | 9 |  | 15 |  | 0 |
|  | ORF1b: L749L |  | 19 |  | 2 |  | 15 |  | 0 |
|  | ORF1b: R1315C |  | 18 |  | 3 |  | 15 |  | 0 |
|  | ORF1b: T2163I |  | 15 |  | 6 |  | 15 |  | 0 |
|  | ORF1b: E2196E |  | 15 |  | 6 |  | 15 |  | 0 |
|  | S: T19I |  | 13 |  | 8 |  | 15 |  | 0 |
|  | S: LPPA24Sdel |  | 12 |  | 9 |  | 15 |  | 0 |
|  | S: V213G |  | 16 |  | 5 |  | 15 |  | 0 |
|  | S: S371F |  | 21 |  | 0 |  | 12 |  | 3 |
|  | S: T376A |  | 20 |  | 1 |  | 15 |  | 0 |
|  | S: D405N |  | 20 |  | 1 |  | 15 |  | 0 |
|  | S: R408S |  | 20 |  | 1 |  | 15 |  | 0 |
|  | ORF3a: T223I |  | 14 |  | 7 |  | 15 |  | 0 |
|  | M: F112F |  | 15 |  | 6 |  | 15 |  | 0 |
|  | ORF6:D61L |  | 15 |  | 6 |  | 15 |  | 0 |
|  | N: S413R |  | 16 |  | 5 |  | 15 |  | 0 |
|  | Omicron BA.2 haplotype |  | 21 |  | 0 |  | 11 |  | 4 |
|  | S: H69^-^/V70^-^ (Sequencing_ | 15-02-2022 to 12-04-2022 | 10 | 15-02-2022 to 12-04-2022 | 1 |  | N/A |  | N/A |
|  | S: H69^-^/V70^-^ (AS-RT-qPCR) |  | 10 |  | 1 |  | N/A |  | N/A |

Figure S1 Spearman correlations between SARS-CoV-2 variants frequency estimations derived through single-allele and haplotype from sequenced wastewater samples. The analysis revealed significant positive correlations between N: D63G and B.1.617.2 haplotype (r = 0.887, *p* < 0.05), N: P13L and B.1.1.529 haplotype (r = 0.860, *p* < 0.05), S: H69^+^/V70^+^ and BA.1 haplotype (r = 0.903, *p* = 0.05), and S: H69^-^/V70^-^ and BA.2 haplotype (r = 0.873, *p* = 0.05). The error bar of each graph represents the uncertainty or relative variation of data points from the fitting line.

**References**

CDC. (2020). Real-Time RT-PCR diagnostic panel for emergency use only. *Cdc Eua*, *3*, 1–42.

D’Aoust, P. M., Tian, X., Towhid, S. T., Xiao, A., Mercier, E., Hegazy, N., et al. (2022). Wastewater to clinical case (WC) ratio of COVID-19 identifies insufficient clinical testing, onset of new variants of concern and population immunity in urban communities. *MedRxiv*, 2022.04.19.22274052.

Peterson, S. W., Lidder, R., Daigle, J., Wonitowy, Q., Dueck, C., Nagasawa, A., et al.. (2022). RT-qPCR detection of SARS-CoV-2 mutations S 69–70 del, S N501Y and N D3L associated with variants of concern in Canadian wastewater samples. *Science of the Total Environment*, *810*, 151283. https://doi.org/10.1016/j.scitotenv.2021.151283
